# Validation of a physical activity scale for older adults participating in the Health and Retirement Study

**DOI:** 10.1093/geroni/igaf122.4232

**Published:** 2025-12-31

**Authors:** Peter Hart

**Affiliations:** Kinesmetrics, Tallahassee, Florida, United States

## Abstract

The aim of this research was to validate a new scale measuring physical activity (PA) using items contained in a large national survey of older adults. Data from 15,335 adults 50+ years of age participating in the 2022 Health and Retirement Study were used. The assessment strategy involved six steps: 1) defining the PA scale (PAS) items and categories, 2) factor analysis, 3) internal consistency reliability, 4) item response theory (IRT) analysis, 5) convergent validity correlations, and 6) modeling PAS scores with a general health (GH) outcome. Polychoric correlations between items were used for the classical analyses. A graded response model (GRM) for polytomous items was employed for the IRT analysis. Multinomial logistic regression was used to model GH categories with PAS scores. The PAS included 3 items of vigorous, moderate, and light PA, each with 3 categories of inactive, low/moderately active, and highly active. Factor analysis retained a single factor with 70% explained variance, whilst the reliability coefficient for items was 0.79. IRT calibration showed category thresholds ranging from -1.90 to 1.05 and item discrimination parameters between 1.39 and 5.00. IRT theta scores correlated with the PAS sum score (r = 0.97), age (r=-0.21), GH (r = 0.38), and timed walk performance (r = 0.35). Modeling showed that for each point increase in PAS score, odds of poor (OR = 0.31, 0.27-0.34), fair (OR = 0.47, 0.43-0.52), good (OR = 0.60, 0.56-0.65), and very good (OR = 0.79, 0.73-0.85) GH, as compared to excellent (reference), continually decreased. These results support the use of a simple 3-item PAS to measure PA in older adults.

